# Protein Synthesis Inhibition in the Peri-Infarct Cortex Slows Motor Recovery in Rats

**DOI:** 10.1371/journal.pone.0157859

**Published:** 2016-06-17

**Authors:** Maximilian Schubring-Giese, Susan Leemburg, Andreas Rüdiger Luft, Jonas Aurel Hosp

**Affiliations:** 1 Division of Vascular Neurology and Rehabilitation, Department of Neurology, University and University Hospital of Zurich, Zurich, Switzerland; 2 cereneo Center for Neurology and Rehabilitation, Vitznau, Switzerland; 3 Department of Neurology, Johns Hopkins University, 1550 Orleans Street, Baltimore, Maryland, 21231, United States of America; Fraunhofer Institute for Cell Therapy and Immunology, GERMANY

## Abstract

Neuroplasticity and reorganization of brain motor networks are thought to enable recovery of motor function after ischemic stroke. Especially in the cortex surrounding the ischemic scar (i.e., peri-infarct cortex), evidence for lasting reorganization has been found at the level of neurons and networks. This reorganization depends on expression of specific genes and subsequent protein synthesis. To test the functional relevance of the peri-infarct cortex for recovery we assessed the effect of protein synthesis inhibition within this region after experimental stroke. Long-Evans rats were trained to perform a skilled-reaching task (SRT) until they reached plateau performance. A photothrombotic stroke was induced in the forelimb representation of the primary motor cortex (M1) contralateral to the trained paw. The SRT was re-trained after stroke while the protein synthesis inhibitor anisomycin (ANI) or saline were injected into the peri-infarct cortex through implanted cannulas. ANI injections reduced protein synthesis within the peri-infarct cortex by 69% and significantly impaired recovery of reaching performance through re-training. Improvement of motor performance within a single training session remained intact, while improvement between training sessions was impaired. ANI injections did not affect infarct size. Thus, protein synthesis inhibition within the peri-infarct cortex impairs recovery of motor deficits after ischemic stroke by interfering with consolidation of motor memory between training sessions but not short-term improvements within one session.

## Introduction

Survivors of ischemic stroke often suffer from motor deficits that recover during days or weeks either spontaneously and/or supported by rehabilitative training. In human stroke-survivors, activation of cortical areas in the vicinity of the ischemic scar, the peri-infarct cortex is associated with a favorable outcome [1].

In rodent models of cortical ischemic stroke, profound structural changes such as axonal sprouting and formation of novel synapses are known to occur in the peri-infarct cortex [2]. These plastic changes critically depend on de novo synthesis of proteins [3, 4]. Protein synthesis inhibition (PSI) through systemic or local application of inhibitors of the peptidyl transferase (e.g. anisomycin, ANI) abolishes memory consolidation by preventing protein-synthesis dependent plastic processes. Injection of ANI into the hippocampus impairs spatial learning [5] and application of ANI into the amygdala interferes with learning in a classical conditioning paradigm [6]. Likewise, acquisition of skilled reaching (SRT) is impaired by ANI-injection into the primary motor cortex [7].

To test the hypothesis that the peri-infarct cortex plays a crucial role for motor recovery, we performed local ANI-mediated PSI in a rat model of photothrombotic stroke within the primary motor cortex.

## Materials and Methods

### Animals and experiments

Adult 9–12 weeks old male Long-Evans rats (n = 51; 200–370 g; Janvier Labs, Le Genest—St-Isle, France) were used for this study. Animals were housed in cages in groups of three individuals in a 12/12-hour light/dark cycle. For all experiments, litter-mates were equally assigned to experimental groups. Six rats had to be euthanized due to perioperative complications and were therefore excluded from the study. Training sessions were performed at the beginning of the dark phase. Animals were food-deprived for 24 hours prior to the first training session. Daily food intake was limited to ca. 50g/kg body weight of standard chow, provided after each training session. Water was available ad libitum. Body weight remained stable throughout the experiment. All experiments were conducted in accordance with Swiss regulations and were approved by the veterinary office of the Canton Zürich.

### Experimental setup and behavioral experiments

**Fig 1:**
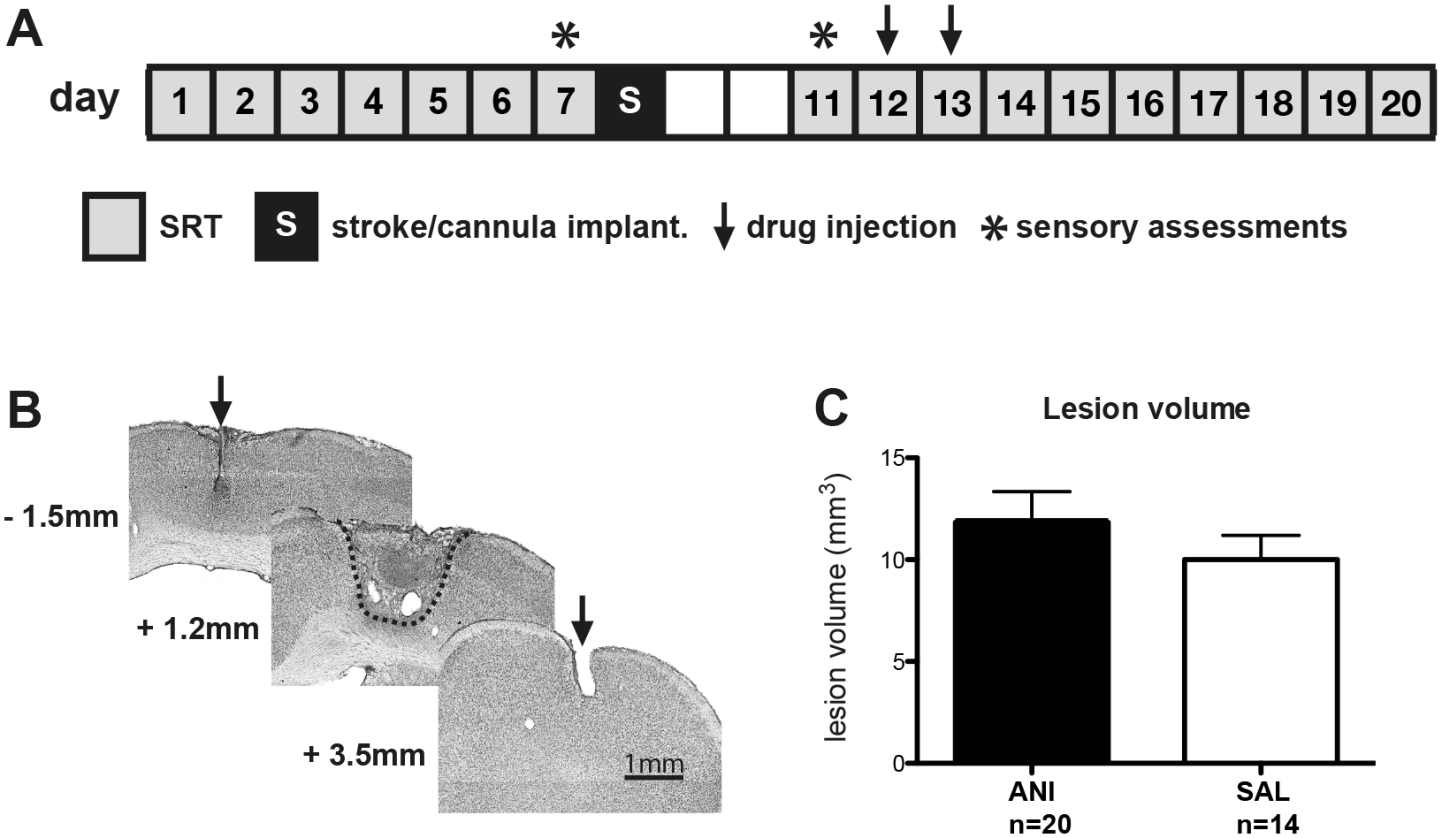
**A** Experimental design indicating the timing of behavioral training and interventions. 20 rats were injected with anisomycin (ANI), 14 rats were injected with saline (SAL). **B** Coronal sections stained with cresyl violet from a representative animal. The arrowheads indicate the placement of injection cannulas and the dashed line indicates the photothrombotic lesion. Longitudinal coordinates refer to bregma. **C** Volumes of photothrombotic strokes are not different between groups. Values represent mean ± SEM.

The experimental protocol is summarized in Fig 1A. The SRT was performed as previously described [8]. Rats were trained to reach and grasp for a food pellet, placed outside a training cage (15 x 40 x 30 cm) with a vertical window (1 cm wide, 5 cm high, lower edge 5 cm above ground) in the front wall and a small light sensor in the rear wall (7 cm above ground).

During a pre-training phase animals learned to open the motorized sliding door that covered the front window by touching a sensor in the rear. Opening the window gave access to a single food pellet that was retrievable by tongue (45 mg, Bio-serve, Frenchtown, NJ, USA).

Reaching training started after animals performed 100 trials in less than 30 minutes on two consecutive pre-training days. Forelimb preference was determined by offering a food pellet in a distance of 15 mm in front of the window. In this position pellets were only retrievable by forelimb reaching. Animals were allowed to perform 20 reaching attempts and the paw that was used most frequently was defined as the preferred side. SRT was initiated by placing the pellet on a small vertical post 15 mm away from the window. This post was shifted to align with the edge of the window, allowing the use of the preferred limb only. Because the diameter of the post was approximately that of the pellet, the pellet was in an unstable position easily knocked off the post. Each reaching attempt (trial) was scored as “successful” (reach, grasp and retrieve) or “unsuccessful”. “Unsuccessful” trials where rats managed to grasp the pellet but dropped it during paw retraction were counted separately as drops. Each training session consisted of 100 trials or 60 minutes, whichever came first. Reaching performance was defined as number of successful trials out of 100 possible trials (success rate). Evolution of reaching performance within a single session was assessed by sampling the number of successful grasps per 20 trials in a bin. The intra-session change was calculated by subtracting the number of successes of the first (trial 1–20) from the last bin (trial 81–100). The performance change between two sessions was calculated by subtracting the number of successes of the first bin (trial 1–20) of the actual from the last bin (trial 81–100) of the preceding training session. The latencies between pellet removal and subsequent door opening were measured to assess operant knowledge. Rats completed seven training sessions ensuring that they had reached a performance plateau before the stroke (days 1–7, Fig 1A). The average success rate of the last three sessions (days 5–7) was used as a measure of plateau performance for each rat. Starting 3 days after stroke, animals were re-trained for ten sessions (days 11–20, Fig 1A). For this rehabilitation phase, the average success rate of the last two sessions (days 19–20) was used as a measure of final performance.

To exclude differences with respect to somatosensory function between groups, we conducted further functional assessments before and after lesioning. For the sticky tape test [9], round sticky labels of 12 mm in diameter (Avery, Zollikofen, Switzerland) were applied to the animal’s forepaws on the palmar side. The time required to remove the label from each forelimb was recorded. For each trial, both paws were tested one after another. Testing sessions consisted of 5 trials per session with a maximum of 3 minutes per removal attempt. To assess the functional forelimb asymmetry the cylinder test was performed [10]. Rats were placed inside a transparent cylinder (30 cm in diameter and 25 cm in height) and evaluated for 3 minutes. The number of cylinder wall touches with each forepaw was counted. Each test was performed on the last training day before the stroke (day 7) and before the first re-training session after stroke (day 11).

For both the sticky tape task and the cylinder task, data was lost for 3 rats in the ANI group due to a computer failure. However, the evolution of re-training was not different in these three animals when compared to those with complete data of sensory assessments (for group effect: F(1) = 0.58, p = 0.46; for interaction of group x time: F(9) = 1.5, p = 0.15). Thus, we are confident that the data loss does not impair the validity of our conclusions.

### Surgical procedures and photothrombotic stroke

A 10 x 5 mm craniotomy contralateral to the preferred paw was performed (position relative to bregma: 6mm anterior, 4mm posterior and 0.5 mm lateral) under ketamine (Ketalar, Bayer; 100 mg/kg body weight, i.p.) and xylazine (Streuli; 10 mg/kg body weight, i.p.) anesthesia with the rats fixated in a computer-controlled stereotaxic instrument (Dual Benchmark Angle One; Harvard Apparatus). Additional ketamine doses (30 mg/kg, i.p.) were administered if necessary. Body temperature was controlled using a heating pad. Buprenorphine (0.01 mg/kg, i.p.) was given after surgery for pain relief.

A photothrombotic stroke was induced as described earlier [11]. In brief, Rose Bengal dye (13μg/g body weight; 10 mg/ml in sterile saline; Sigma Aldrich, Germany) was injected into the tail vein using a motorized pump (Genie, Kent Scientific Corporation, Torrington, USA) during the first 2 minutes of a 20-minute illumination period using a cold light source (KL 1500, Schott AG, Mainz, Germany). To restrict lesion size, a 2-millimeter diameter aluminium foil stencil was centered on the forelimb area of the primary motor cortex (1 mm anterior and 3 mm lateral, relative to bregma). Subsequently, two guide cannulas (30 ga, 15 mm long, Unimed SA, Lausanne, Switzerland) were inserted to a depth of 900 μm into the peri-infarct cortex rostral and caudal to the ischemic lesion (positions relative to bregma: 3.5 mm anterior and 3 mm lateral and -1.5 mm posterior and 3 mm lateral). Finally, the skull was reconstructed using the bone flap derived from the initial craniotomy and dental cement (Flowline, Heraeus Kulzer GmbH, Hanau, Germany). A bone screw anchored in the occipital contralesional skull for a ensured a stable implant. After surgery, animals were returned to their home cages for a 3-day recovery period.

All drug injections were given into both cannulas using 34 ga, 15 mm long needles (Unimed SA, Lausanne, Switzerland). Rats were sedated briefly with isoflurane (duration < 2 min) and injected with 1 μl per cannula of anisomycine in saline (ANI, 100 μg/μl, Sigma-Aldrich; n = 21) or with saline alone (SAL, n = 15) using a 5μl microsyringe (Hamilton, Bonaduz, Switzerland) and a motorized pump with the injection speed of 1 μl/min (Stereotaxic microinjection pump, Stoelting, Wood Dale, IL, USA). The drug injections were performed on second and third retraining session after stroke (day 12 and 13, Fig 1A). Injections were performed immediately after training to avoid an impairment of training due to isoflurane exposure. The time elapsed between the final trial of a retraining session and injection was shorter than 5 minutes for all rats. Rats were randomly allocated to groups (ANI or SAL) by drawing lots. Drug application was performed in a blinded manner and researchers performing behavioral experiments, histological examinations and evaluation of protein synthesis were not aware of group identities.

### Evaluation of protein synthesis inhibition (PSI)

The level of PSI after ANI injection was assessed via incorporation of ^35^S-labeled methionine (Amersham Biosciences, Freiburg, Germany) into newly synthesized proteins. For this control experiment, a timeline different from that depicted in Fig 1A was applied: eleven rats received a photothrombotic stroke followed by implantation of cannulas into the peri-infarct cortex as described above. After three days of recovery, animals were injected with ANI (n = 6) or saline (n = 5). Two hours after these injections, ^35^S-methionine (1 μl of 10 μCi/μl in each cannula; 1 μl/min) was given. Three hours after ^35^S-methionine injection, animals were killed with an overdose of pentobarbital (50mg/kg i.p.; Kantonsapotheke Zurich, Switzerland), and the peri-infarct cortex was dissected. Tissue samples were weighed and homogenized in 1.5 ml of lysis buffer per 100 mg of tissue (1% SDS in Tris-HCl, pH 8.5) in the presence of protease inhibitors (cOmplete ULTRA mini, Roche, Switzerland). The homogenate was boiled for 5 min at 95°C and centrifuged (10.000 rpm for 15 min at 4°C). Protein precipitation was achieved by incubating the supernatant with icecold trichloroacetic acid (TCA, 25% final concentration) for 60 min and centrifugation (13.000 rpm for 20 min at 4°C). A scintillation counter was used to measure radioactivity of the protein pellet and of the supernatant. For each animal, a pellet-to-supernatant index was computed as the ratio of pellet scintillation counts to supernatant counts.

### Histology and stroke volume analysis

The infarct volume was assessed histologically (Fig 1B). After the final training session on day 20, rats were killed using an overdose of pentobarbital (50 mg/kg i.p.; Kantonsapotheke Zurich, Switzerland) and perfused transcardially with 0.1M phosphate-buffered saline (PBS) followed by 4% paraformaldehyde (PFA) in PBS. Brains were extracted and cryoprotected in 20% sucrose in PBS for 2–5 days and subsequently frozen at –80°C. 50-μm-thick coronal sections were prepared using a cryostat (Leica Microsystems GmbH, Wetzlar, Germany), stained with cresyl violet (Sigma Aldrich, Germany) and mounted with Permount mounting medium (bioWorld, Dublin, Ohio, USA). Every tenth section was used for measurement of lesion size. Brain sections were digitized at 5x magnification using a microscope (Axioplan II, Germany with Axio Cam MR, Zeiss, Germany), the lesioned area and cannula tracts were measured and reconstructed using ImageJ software (NIH, http://imagej.nih.gov/ij/). Lesioned tissue was identified by lack of neurons and the presence of glial scar tissue. Lesion volume was computed by multiplying the total measured lesion area for each animal by the distance between sections (500 μm). All cannulas were implanted with an average distance of 0.56 ± 0.09 mm to the edge of the ischemic scar.

### Statistical analysis

Statistical analyses were performed using Prism 5.0 (GraphPad Inc., San Diego, CA, USA) and SPSS Statistics 22.0 (IBM Corp., Armonk, NY, United States). Learning and recovery curves, as well as sticky tape performance and cylinder test were compared using repeated measures ANOVA, with factors group (ANI vs. SAL) and time. The sphericity assumption was tested using the Mauchly criterion and the Greenhouse-Geisser correction was used where appropriate. For learning and recovery curves, performance during the first session (day 1 or day 11 respectively) was added as a covariate to avoid false-positive results caused by baseline differences.

Lesion volume, reaching performance at plateau and drop of reaching performance after stroke were compared using independent-samples t-tests, after normal distribution was assessed using the Shapiro-Wilk test. ^35^S-methionin-incorporation was compared using the non-parametric Kruskal-Wallis test due to non-normal distribution of the data. Results are expressed as mean ± standard error of the mean (SEM).

## Results

Injection of ANI into the peri-infarct cortex reduced protein synthesis by 69% compared to SAL injection (pellet scintillation count/supernatant count: 2.3 ± 0.7 for ANI, N = 6 vs. 7.2 ± 1.9 for SAL, N = 5; p = 0.017). Ischemic lesion volume was not affected by PSI (11.8 ± 1.5 mm^3^ for ANI N = 20 vs. 10.1 ± 1.2 mm^3^ for SAL, N = 14; t(df) = 0.9(32); p = 0.37; Fig 1C).

**Fig 2:**
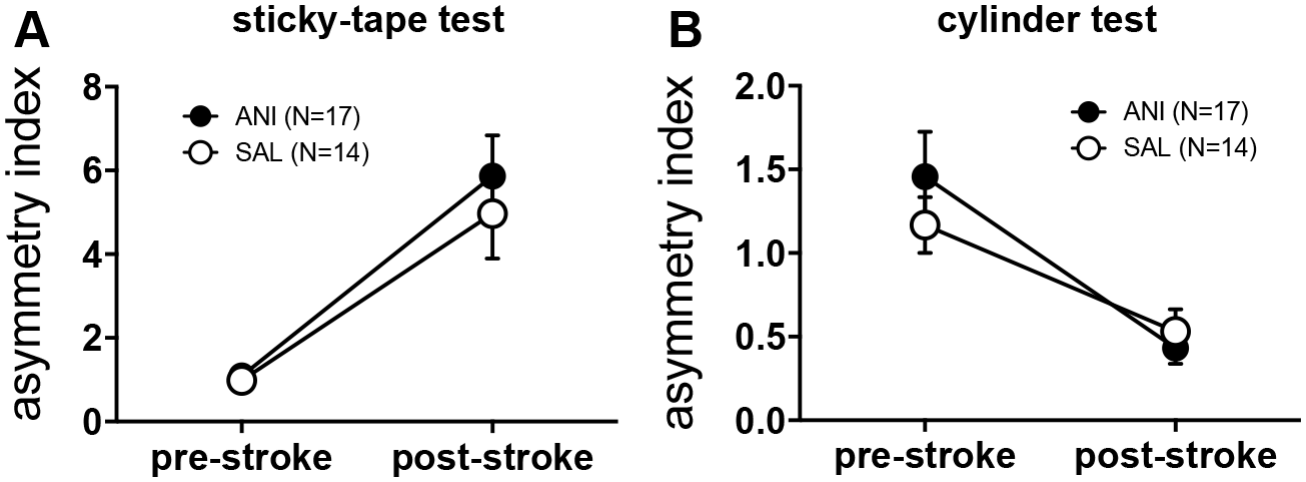
**A** The sticky tape test reveals a significant sensory deficit for the contralesional paw resulting in an increased asymmetry index (time to remove tape from contralesional vs. ipsilesional paw). Sensory deficits were not different in ANI and SAL animals. **B** In the cylinder test, spontaneous use of the ipsilesional paw decreased in favour of the contralesional paw, resulting in a decreased asymmetry index (number of touches contralesional vs. ipsilesional). There was no difference between ANI and SAL animals. All values are presented as mean ± SEM.

The stroke caused a significant sensory deficit in the sticky tape test performance in both SAL and ANI animals (N = 17 for ANI, N = 14 for SAL; repeated measures ANOVA: mean effect of time: F(1,29) = 37.1, p<0.0001; mean effect of group: F(1,29) = 0.46, p = 0.5; group x time F(1,29) = 0.3, p = 0.59; Fig 2A). Spontaneous forelimb use in the cylinder test, was not affected by the stroke (N = 17 for ANI, N = 14 for SAL; repeated measures ANOVA: mean effect of time: F(1,29) = 30.9, p<0.0001; mean effect of group: F(1,29) = 0.23, p = 0.63; group x time F(1,29) = 1.7, p = 0.3; Fig 2C). (N = 17 for ANI, N = 14 for SAL; mean effect of time F(1) = 1.3, p = 0.26; effect of group x time F(1) = 0.59, p = 0.45; Fig). However, use of the contralesional paw was reduced in favor of use of the ipsilesional paw in both groups, as indicated by a significant reduction in the asymmetry index (N = 17 for ANI, N = 14 for SAL; repeated measures ANOVA: mean effect of time: F(1,29) = 30.9, p<0.0001; mean effect of group: F(1,29) = 0.23, p = 0.63; group x time F(1,29) = 1.7, p = 0.3; Fig 2C).

All animals acquired the skilled reaching task without differences between groups (N = 20 for ANI, N = 14 for SAL; repeated measures ANOVA: main effect of group: F(1) = 0.1, p = 0.42; effect of group x time: F(4.1) = 0.59, p = 0.675). Pre-stroke plateau reaching performance was not significantly different between groups (success rate of 31.2 ± 2.7% for ANI vs. 27.6 ± 2.2% for SAL; t(df) = 0.96(32); p = 0.35). Both groups showed an equal drop in reaching performance after stroke (reduction in success rate from day 7 to day 11: 48.2 ± 10.5% for ANI vs. 48.4 ± 12% for SAL; t(df) = 0.007(32); p = 0.99), indicating a similar motor deficit. As a correlate of the somatosensory deficits, number of drops (i.e. reaching attempts where rats were able to grasp the pellet but dropped it during paw retraction) significantly increased after stroke equally in both groups (repeated measures ANOVA of drops from day 7 to day 11: mean effect of time: F(1) = 16.5, p = 0.001; mean effect of group: F(1) = 0.02, p = 0.89; interaction of group x time: F(1) = 1.2, p = 0.29).

**Fig 3:**
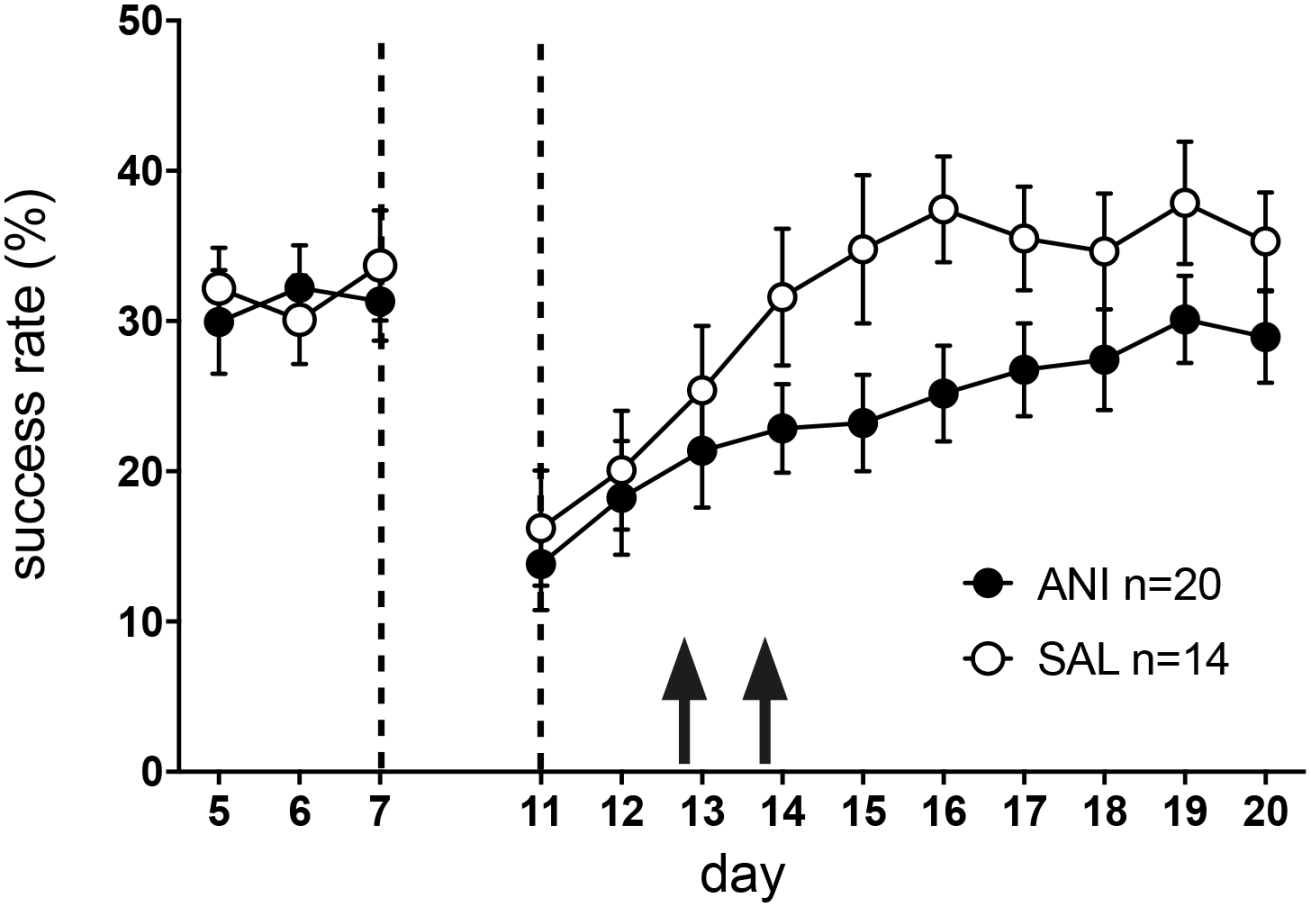
Both groups acquired the skilled reaching task and achieved a comparable level of performance. After stroke, both groups showed a similar drop in performance of approximately 50%. Injection of ANI (closed circles) into the peri-infarct cortex at day 12 and 13 induced an impairment of recovery when compared to injections of saline (open circles). Values are presented as mean ± SEM.

Injection of ANI (N = 20) into the peri-infarct cortex after the second and third re-training session (day 12 and 13) impaired recovery compared to saline injections (N = 14; repeated measures ANOVA: main effect of group: (F(1, 34) = 5.7, p = 0.024; Fig 3A). This impairment was transient and ANI animals approximate performance plateau of SAL animals on day 19 (success rate 28.7 ± 2.6% for ANI vs. 35.7 ± 3.4% for SAL; t(df) = 1.6(32), p = 0.13). Lesion volume and pre-stroke plateau reaching performance were initially included as independent variables but were removed after they showed no significant effects. ANI injections did not affect operant knowledge as inter-trial latencies were not different between groups (repeated measures ANOVA: mean effect of group: F(1) = 1.0, p = 0.32; interaction of group x time: F(9) = 0.5, p = 0.87). Likewise, ANI did not influence the number of pellet drops during re-training (repeated measures ANOVA day 11–20: mean effect of group: F(1) = 0.01, p = 0.02; interaction of group x time: F(9) = 0.27, p = 0.98), consistent with a similar somatosensory deficit in both groups. For both groups, plateau performance after stroke did not differ from pre-stroke performance (for ANI pre-stroke: 31.2 ± 2.7% vs. post-stroke: 29.5 ± 2.8%, p = 0.62; for SAL pre-stroke: 27.6 ± 2.2% vs. post-stroke: 35.7 ± 3.4%; p = 0.13).

**Fig 4:**
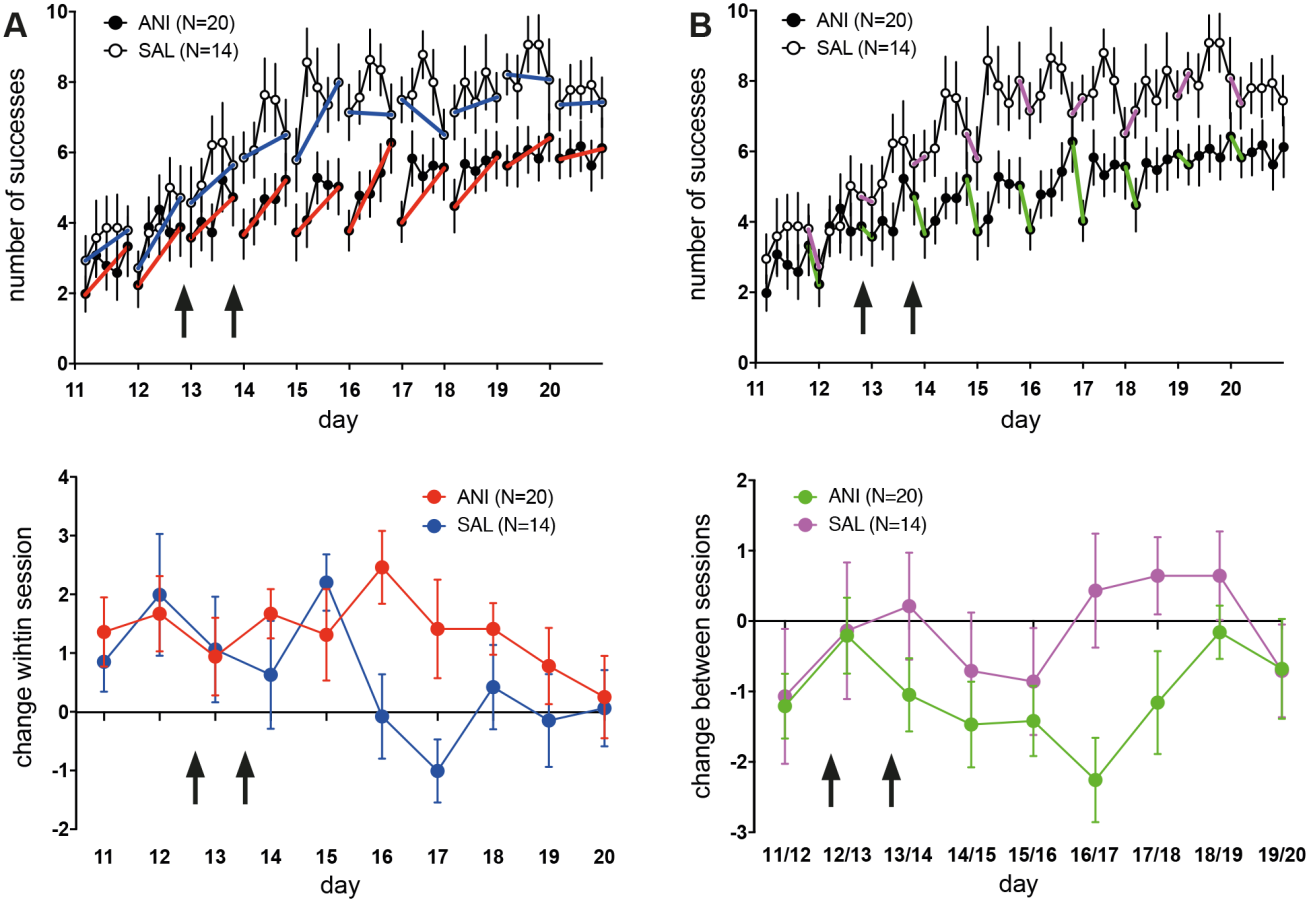
**A top and bottom** To assess intra-session improvement of grasping performance, sessions were divided into 20-trial bins. The percentage of successful grasps is plotted per bin. Intra-session improvement is indicated by blue (SAL) and red lines (ANI). Injection of ANI (filled circles) did not interfere with intra-session improvement consistent with a preserved ability for short-term plasticity. Changes in motor performance within session occur until day 19 after injection of ANI. In contrast, within-session changes drop to zero in SAL animals when they reach plateau on day 16. Values are presented as mean ± SEM. Arrows indicate the time-point of injections. Values represent mean ± SEM. **B top and bottom** The magnitude of decrease in performance between the end of a training session and the start of the subsequent session is inversely correlated with the efficacy of motor memory retention. For SAL animals (open circles), retention between remains largely stable throughout the recovery period as indicated by magenta lines. In ANI rats, retention gets worse after ANI injections are started and normalize after session 18 and 19. Values are presented as mean ± SEM. Arrows indicate the time-point of injections. Values represent mean ± SEM.

Injecting ANI after session 2 and 3 of re-training (days 12 and 13) did not interfere with intra-session improvement (Fig 4A top), indicating a preserved ability for short-term learning. With respect to intra-session change (Fig 4A bottom), a strong but non-significant trend exists for a difference between groups (repeated measures ANOVA: main effect of group: F(1) = 3.3,; p = 0.079; main effect of time: F(6.59) = 1.54; p = 0.16; group x time: F(6.59) = 1.36; p = 0.23). SAL animals reached plateau performance at day 15, after which no further intra-session learning occurred (Fig 4B top). For the ANI-group, intra-session improvement was observed until day 19. With respect of performance-change between sessions (Fig 4B bottom), a significant difference exists between groups (repeated measures ANOVA: main effect of group: F(1) = 4.5,; p = 0.042; main effect of time: F(8) = 1.23; p = 0.28; group x time: F(8) = 1.0; p = 0.43). Thus, inhibition of protein synthesis within the peri-infarct cortex interferes with between-session but not intra-session learning.

## Discussion

*De novo* learning of the skilled reaching task critically depends on the integrity and functionality of the primary motor cortex [7, 12, 13], a brain region that is considered as the key structure where motor memories are stored under physiological conditions [2, 12, 14]. In the photothrombotic stroke model used here, the M1 forelimb representation contralateral to the rat's preferred paw is destroyed and reaching performance drops to 50% of pre-stroke performance. Rats are able to regain reaching performance, a process that is supported by continuously practicing the SRT as a rehabilitative training [15, 16].

Although the structures involved in this recovery process are poorly understood, the peri-infarct region may adopt lost function during recovery [1]. The peri-infarct cortex is defined as the area surrounding the ischemic scar and has a width of up to one millimeter in rodent models of focal ischemia [17]. Within this zone, many large-scale and small-scale structural changes occur after stroke. After an initial decrease in spine density, spine turnover becomes elevated for two weeks [18]. Furthermore, dendritic trees within the peri-infarct cortex are extensively remodeled although the amount of change decreases the farther the neurons are located from the infarct border. Due to inflammatory processes, growth-inhibitory peri-neuronal networks within the extracellular matrix degrade [19]. Expression of inhibitory extracellular matrix proteins such as tenascin and chondroitin sulfate proteoglycans, myelin-associated proteins (e.g. NogoA) and growth-cone inhibitory proteins like ephrin and semaphorin class proteins is suppressed and expression of growth-promoting proteins (e.g. inductors of axon growth-cone) is increased, leading to a growth-promoting environment surrounding the infarct [20]. Newly generated axonal fibers can be detected within the peri-infarct cortex three weeks after stroke [21]. Moreover, enhanced long-term potentiation (LTP) has been detected surrounding the ischemic lesion [22]. In this study, we prevented protein synthesis within the peri-infarct cortex by injecting anisomycin during re-training of the SRT after a photothrombotic stroke. Protein synthesis is thought to be essential for plastic changes that occur within the peri-infarct cortex, like axon growth and dendritic plasticity [3, 4]. By showing that protein synthesis inhibition slows re-learning the SRT, we provide further evidence for a role of the peri-infarct cortex in motor function recovery within the reorganizing brain after ischemic injury.

While recovery is impaired by PSI in the peri-infarct cortex, it is not completely abolished (Fig 3A). This is expected, as post-stroke plasticity also occurs in brain areas outside the peri-infarct cortex. Rehabilitative training after a motorcortical stroke leads to a decreased number of inhibitory interneurons within the premotor area [23] in mice. In squirrel monkeys that received a stroke within the hand representation of M1, intracortical microstimulation (ICMS) mapping revealed an enlargement of hand representations in the ventral premotor cortex [24] and the supplementary motor cortex [25]. Likewise, functional magnetic resonance imaging (fMRI) studies in human stroke survivors suggest a role for both regions motor and pre-motor regions in promoting recovery [26] Furthermore, post-stroke reorganization may also occur within the contralesional hemisphere. In rodents, rehabilitative training after stroke increasesd dendritic length of layer V pyramidal neurons within the contralesional, undamaged motor cortex [15] and lidocain-inactivation of the contralesional hemisphere impaired performance of the paw originally affected by stroke [27]. In humans, fMRI revealed enhanced activation of the contralesional hemisphere that is strongest during the first days after stroke [28]. Thus, apart from the peri-infarct cortex, plastic changes in remote areas such as the premotor and supplementary motor cortex, as well as the contralesional hemisphere may enable a certain degree of recovery.

The ANI-induced impairment of motor recovery after stroke seems similar to the effect of ANI-injection into M1 on *de novo* motor learning in healthy rats [7]. However, recovery and *de novo* learning are not identical processes and results from motor learning models cannot be automatically transferred to post-stroke recovery. While important at certain times during recovery, learning mechanisms are not necessarily driving the restitution of motor function in the acute stage when the stroke has induced a critical period of heightened plasticity [20, 29]. We have previously shown that, in the same photothrombotic stroke model used in the current study, learning curves of rats that newly learned the SRT after stroke are different from those of rats that were trained prior to stroke and were retrained after stroke [11].

Despite these obvious differences, recovery and *de novo* learning share further commonalities beyond their dependency of protein synthesis: while retention of motor performance between sessions was impaired during recovery, PSI did not interfere with the improvement of motor performance within a training session (Fig 4A and 4B). This is similar to the effect of anisomycin on *de novo* learning of the SRT or rotarod running [7, 30] in healthy rats. While formation of novel proteins is required for longer lasting structural changes [31] and alterations of synaptic weights such as LTP-formation [32], within-session improvements may depend on proteins that are already synthesized and present at the synaptic site [33].

The impairment in retention of motor memory between sessions does not normalize until session 18 and 19 (Fig 4A and 4B) although ANI was injected only at day 12 and 13. On the one hand, this can be explained by the long-lasting effect of ANI—the capacity for protein synthesis is only fully restored 48 hours after an its injection into cortex [7]. On the other hand, PSI could have led to a depletion of proteins that are required for consolidation and are only slowly restocked after protein synthesis is re-established. After the effect of ANI dissipates, ANI-injected animals approximate the performance level of SAL-injected rats. In rats, rehabilitation training can effectively support motor recovery towards a normal level of performance within a time frame of two to four weeks stroke [34]. As ANI was injected shortly after stroke in this study, animals should still retain the capability for full recovery.

As protein synthesis is an energy-consuming process [35], reduction of translation may save energy that can be alternatively used to protect cellular integrity and housekeeping functions under the condition of low oxygen availability. In line with this hypothesis, application of anisomycin reduces hypoxic neuronal cell damage in vitro [36, 37]. Protein synthesis inhibition may thus have neuroprotective effects [38]. In the current study, PSI did not influence the size of ischemic infarct. However, anisomycin was injected locally in the peri-infarct for two days, starting five days after stroke. At this time, the ischemic infarct has largely stabilized and the neuroprotective treatment has little effect [39]. Thus, duration and extent of PSI might have been not sufficient to obtain a significant effect on infarct volume. However, as PSI within the peri-infarct cortex slowed motor recovery, prevention of protein synthesis dependent plasticity seems to overbalance a potential neuroprotective effect of anisomycin.

The peri-infarct cortex in rats has a width of approximately one millimeter [17]. Cannulas were implanted within 0.56 ± 0.09 mm of the edge of the ischemic scar, ensuring a precise administration of anisomycin. However, injected fluids spread up to two millimeters from the injection point [7]. Thus, a certain degree of PSI outside of the peri-infarct cortex can be expected. Based on the location of the cannulas, anisomycin could spread into the rostral motor area (RMA), prefrontal cortex (PFC) and somatosensory cortex. ANI-related impairments in these areas could contribute to the reported impairments in re-learning the SRT after stroke. We recorded inter-trial latencies as a measure of operant knowledge of non-motor aspects of the SRT. Reduced PFC function results in a severe deficit of attention and working memory [40], whereas an inhibition of the RMA is thought to interfere with action selection [41]. Both would likely have lead to increased inter-trial latencies in the ANI group. As this was not the case, anisomycin does not seem to have a major effect on these areas. We tested somatosensory function for both groups before and after stroke, but not after the administration of anisomycin. However, number of dropped pellets was not different in both groups, arguing against a severe impairment of sensory function in response to ANI injections.

In this study, we show that protein synthesis inhibition (PSI) within the peri-infarct cortex induces a significant slowing of motor recovery after photothrombotic stroke in rats. This slowing is caused by impaired retention of motor skill between training sessions but not short-term improvements within a training session. These results show that the peri-infarct cortex plays a functional role for motor recovery within the reorganizing brain after ischemic injury. Furthermore, the requirement for protein synthesis may be seen as evidence for local neuroplasticity processes mediating improvement of post-stroke motor performance. This requirement is shared by healthy learning.
